# Developmental Dynamics of General and School-Subject-Specific Components of Academic Self-Concept, Academic Interest, and Academic Anxiety

**DOI:** 10.3389/fpsyg.2016.00356

**Published:** 2016-03-17

**Authors:** Katarzyna Gogol, Martin Brunner, Franzis Preckel, Thomas Goetz, Romain Martin

**Affiliations:** ^1^Department of Education and Psychology, Free University of BerlinBerlin, Germany; ^2^Berlin-Brandenburg Institute for School QualityBerlin, Germany; ^3^Department of Psychology, University of TrierTrier, Germany; ^4^Department of Empirical Educational Research, University of KonstanzKonstanz, Germany; ^5^Department of Empirical Educational Research, Thurgau University of Teacher EducationKreuzlingen, Switzerland; ^6^Luxembourg Centre for Educational Testing, Faculty of Language and Literature, Humanities, Arts and Education, University of LuxembourgLuxembourg, Luxembourg

**Keywords:** academic self-concept, academic interest, academic anxiety, development, nested-factor model

## Abstract

The present study investigated the developmental dynamics of general and subject-specific (i.e., mathematics, French, and German) components of students' academic self-concept, anxiety, and interest. To this end, the authors integrated three lines of research: (a) hierarchical and multidimensional approaches to the conceptualization of each construct, (b) longitudinal analyses of bottom-up and top-down developmental processes across hierarchical levels, and (c) developmental processes across subjects. The data stemmed from two longitudinal large-scale samples (*N* = 3498 and *N* = 3863) of students attending Grades 7 and 9 in Luxembourgish schools. Nested-factor models were applied to represent each construct at each grade level. The analyses demonstrated that several characteristics were shared across constructs. All constructs were multidimensional in nature with respect to the different subjects, showed a hierarchical organization with a general component at the apex of the hierarchy, and had a strong separation between the subject-specific components at both grade levels. Further, all constructs showed moderate differential stabilities at both the general (0.42 < *r* < 0.55) and subject-specific levels (0.45 < *r* < 0.73). Further, little evidence was found for top-down or bottom-up developmental processes. Rather, general and subject-specific components in Grade 9 proved to be primarily a function of the corresponding components in Grade 7. Finally, change in several subject-specific components could be explained by negative effects across subjects.

## Introduction

Academic self-concept, (individual) academic interest, and academic anxiety are key affective-motivational constructs in educational research that have not only been shown to determine academic effort, choices, and success but are also considered to be vital learning outcomes themselves (e.g., Marsh and Yeung, 1997a,b; Zeidner, 1998; Marsh et al., 2005; Marsh and O'Mara, 2008; Goetz et al., 2010; Schunk et al., 2010). Given their relevance for students' learning and educational careers, it is important to understand the developmental dynamics of these constructs. To this end, this article brings together important streams of research that have rarely been integrated before. A particularly important issue for investigations of academic affect or motivation has always been the hierarchical level of construct definitions. Earlier research on academic affect and motivation focused on general constructs (at the top of the hierarchy; e.g., Byrne, 1986) with items such as “I am good at most school subjects.” On the other hand, contemporary educational research has stressed the importance of differentiating between different subjects (e.g., “I am good at mathematics”) with a focus on the lower levels of the construct hierarchy (e.g., Marsh, 1990; Bong, 2001; Goetz et al., 2007). Please note that the term “subject” is used throughout this study instead of the more precise term “school subject” for the clarity of the presentation. Crucially, students differ and develop in their school-related affect and motivation both in general and with respect to specific subjects. However, most research on the development of affective-motivational constructs has focused on either their general or subject-specific level but has not simultaneously accounted for the general and subject-specific components of the constructs from the perspective of a hierarchical construct definition. Thus, there is a limited amount of empirical knowledge about the manifold developmental dynamics of general and subject-specific components of affective-motivational constructs, and several questions have yet to be answered about them: (a) How stable are general and subject-specific components across time? (b) Is the development of affective-motivational constructs characterized by top-down (e.g., Does general academic anxiety affect the development of anxiety in mathematics?) or bottom-up processes (e.g., Does anxiety in mathematics affects the development of general academic anxiety?)? (c) Are there developmental processes across subjects (e.g., Does anxiety in mathematics affect the development of anxiety in verbal subjects?)? To address these research questions, we capitalized on two representative, large-scale data sets and contemporary measurement models to examine the developmental dynamics of general and subject-specific components (i.e., German, French, and mathematics) of academic self-concept, interest, and anxiety, respectively. By doing so, we were able to scrutinize the similarities and differences in the developmental dynamics of these constructs.

### Structure of affective-motivational constructs

Academic self-concepts are mental representations of a person's abilities in subjects (Brunner et al., 2010) entailing aspects of both self-description and self-evaluation (Marsh and Craven, 1997; Brunner et al., 2009). Academic interest comprises feelings of personal importance and emotional value (Schiefele, 1991; Renninger, 2000; Krapp, 2002). In the present study, we refer to academic interest as an individual interest (i.e., a relatively enduring preference for a certain subject) and not as a situational interest (i.e., a current situationally triggered engagement; see Schiefele, 1991). Academic anxiety refers to feelings of worry as well as nervousness and uneasiness in achievement-related situations in the school context (Liebert and Morris, 1967; Zeidner, 2007; Goetz et al., 2008b).

**Figure 1:**
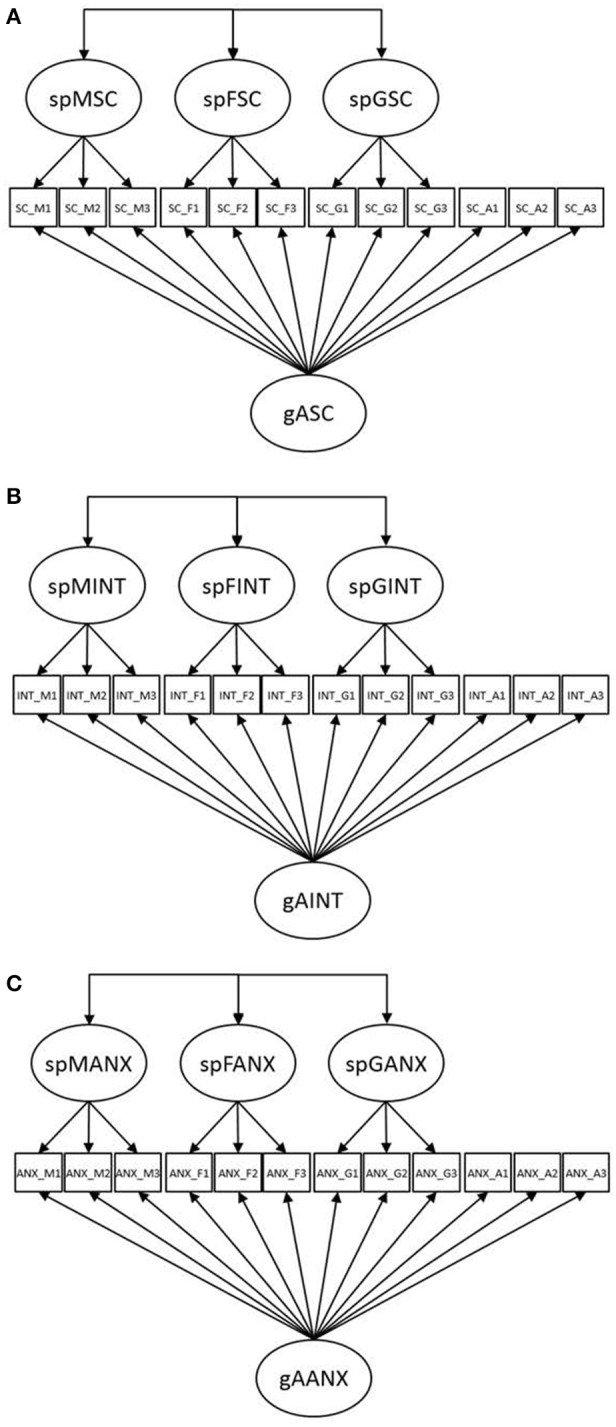
**Schematic diagrams of the nested-factor models for (A) academic self-concept (nested Marsh/Shavelson model; Brunner et al., 2010), (B) academic interest, and (C) academic anxiety as applied in the present study**. Residuals as well as the correlations between the residuals of items with parallel wording are not depicted in the models to ensure the clarity of the figure. gASC = general academic self-concept; spMSC = specific mathematics self-concept; spFSC = specific French self-concept; spGSC = specific German self-concept; gAINT = general academic interest; spMINT = specific mathematics interest; spFINT = specific French interest; spGINT = specific German interest; gAANX = general academic anxiety; spMANX = specific mathematics anxiety; spFANX = specific French anxiety; spGANX = specific German anxiety.

Previous research has strongly supported the multidimensionality of these affective-motivational constructs with respect to subjects (e.g., Marsh et al., 1988; Marsh, 1990; Bong, 2001; Goetz et al., 2007; Brunner et al., 2010). Moreover, not only do students differentiate between different subjects when evaluating their affect and motivation in school, but they also evaluate their overall levels of affective-motivational constructs. Thus, current structural models of academic self-concept conceive of academic self-concept as a construct that is not only subject-specific by nature but also hierarchically organized with general academic self-concept operating at the apex of the hierarchy (see Brunner et al., 2010). Figure 1 depicts the nested Marsh/Shavelson (NMS) model, which has been shown to nicely capture the multidimensional and hierarchical structure of academic self-concepts in representative large-scale studies (Brunner et al., 2008, 2009, 2010; Gogol et al. submitted). In particular, this model specifies a latent variable for general academic self-concept (gASC) that directly influences the general and subject-specific measures of academic self-concept. This specification implies that gASC is the most general construct in the NMS model, an idea that, in turn, is consistent with the idea that gASC operates at the apex of the hierarchy of academic self-concept. Moreover, to represent the multidimensional nature of academic self-concept with respect to specific subjects, the model specifies latent variables that influence corresponding measures of subject-specific self-concepts over and above gASC. Thus, these latent variables represent academic self-concepts that are specific to different subjects [e.g., specific mathematics self-concept (spMSC), specific French self-concept (spFSC), and specific German self-concept (spGSC)]. Crucially, as these subject-specific factors are conceptualized as uncorrelated with the general academic self-concept factor, the general academic self-concept factor controls for the general level of academic self-concept in the measures of subject-specific self-concept. The latent variables representing subject-specific self-concepts thus depict how students perceive their subject-specific strengths/weaknesses over and above their individual general level of self-concept. Moreover, the nested Marsh/Shavelson model does not specify any constraints on the correlational pattern between these subject-specific self-concepts. In previous studies, negative correlations have been found between spMSC, spFSC, and spGSC (Brunner et al., 2010; Gogol et al. submitted), indicating a strong separation of self-concepts across different subjects. Specifically, such negative correlations between subject-specific self-concepts reflect the notion that students think of themselves, for example, as being good in mathematics but not in German, good in mathematics but not in French, or good in German but not in French (see also Marsh and Hau, 2004, p. 57).

Regarding academic interest and academic anxiety, general and subject-specific conceptualizations seem to coexist in the literature. Specifically, some scholars conceive of academic interest as a strongly subject-specific construct (e.g., Schiefele, 1991; Krapp, 2002; Hidi and Renninger, 2006). However, it has also been argued that besides defining interest in terms of specific subjects, students may have a general individual interest in learning (Ainley et al., 2002). Moreover, in more recent educational research (dating back across the last 10–15 years), academic anxiety has been considered to be specific to subjects (Goetz et al., 2007). Yet, the general nature of academic anxiety was emphasized in earlier research (Zeidner, 1998).

The idea of conceiving of both academic anxiety and interest as both hierarchical and multidimensional constructs, however, had not been examined until recently. Specifically, Gogol et al. (2015, submitted) developed and tested new structural models for academic interest and academic anxiety, respectively. The models were specified as analogous to the nested Marsh/Shavelson model of academic self-concept (Brunner et al., 2010). Their results demonstrated that academic interest and anxiety were structurally similar to academic self-concept (Gogol et al., submitted; Gogol et al., 2015; see Figures 1B,C). First, these constructs were shown to have a hierarchical structure with general academic interest (anxiety) at the apex of the hierarchy, indicating that students perceived themselves as generally more interested or less interested (more anxious or less anxious) than other students across different subjects. Second, academic interest (anxiety) was found to be multidimensional with respect to different subjects. In other words, after controlling for students' overall level of interest (anxiety), they differed across different subjects in their perceived interests (anxieties). Third, academic interest (academic anxiety) showed a strong separation between its subject-specific components, indicating that a higher interest (anxiety) in mathematics was associated with lower interests (anxieties) in subjects from the verbal domain. Similarly, a higher interest (anxiety) in French was found to be associated with a lower interest (anxiety) in German. To sum up, the study by Gogol et al. provided strong empirical support for the hierarchical and subject-specific organization of academic interest and academic anxiety, respectively.

### Differential stabilities

In developmental research, construct stability refers to the mean level of stability and differential stability in the rank ordering of individuals. Given that the present study is an investigation of individual development, we chose to focus on differential stability, which is typically analyzed in terms of the correlation of a construct measured at two different occasions (i.e., autocorrelation). Low differential stability is indicated by change in individuals' relative positions within a reference group across time. Conversely, when students retain their ranks with respect to their construct levels within a given student group, differential stability is high.

Previous studies have reported moderate to relatively high stability coefficients in adolescent students for academic self-concept (e.g., Shavelson and Bolus, 1982; Eccles et al., 1989; Marsh et al., 2005; Frenzel et al., 2006; Möller et al., 2011; Pinxten et al., 2014; Musu-Gillette et al., 2015; Parker et al., 2015), academic interest (e.g., Watt, 2000; Köller et al., 2001; Marsh et al., 2005; Frenzel et al., 2006, 2010; Musu-Gillette et al., 2015), intrinsic motivation (a construct that is conceptually close to academic interest; e.g., Gottfried et al., 2001), and enjoyment (the emotional component of academic interest; Pinxten et al., 2014) in mathematics or verbal subjects. For example, Frenzel et al. (2010) found 1-year stabilities in interest in mathematics ranging from 0.54 to 0.65 between Grades 6 and 9. With regard to academic anxiety, there has not been much research on differential stability in adolescents. The existing studies found stability coefficients similar to those for academic self-concept and interest (e.g., Frenzel et al., 2006; Selkirk et al., 2011; Ahmed et al., 2012). However, little is known about the stability of the general level of affective-motivational constructs. For example, theory predicts decreasing stability in self- concept from the apex of the hierarchy (Shavelson et al., 1976) to the lower hierarchical levels, suggesting that general academic self-concept should be more stable than subject-specific self-concepts. Yet, the few previous studies that examined this idea found little support for increases in stability when approaching the apex of the academic self-concept hierarchy (Shavelson and Bolus, 1982; Marsh and Yeung, 1998).

### Prediction of change

Regarding top-down and bottom-up processes, in 1998, Marsh and Yeung published a pioneering self-concept article that is still unique today. As Marsh and Yeung noticed, theoretical considerations of the direction of causal flow in the self-concept hierarchy have been contradictory. Specifically, the Shavelson model of self-concept (Shavelson et al., 1976) and Rosenberg (1979) and Harter's (1986) theoretical considerations implied a bottom-up model in which the direction of causal influence is from the bottom to the top of the hierarchy. On the other hand, Brown (1993) advocated for a top-down model in which the direction of the causal flow is from the apex to the base of the hierarchy. However, these theoretical predictions could not be tested without the appropriate methodology (Marsh and Yeung, 1998). Marsh and Yeung noted that the direction of causal influence could not be determined on the basis of data from only one time point. Thus, in their study, they investigated the direction of causal flow between general and subject-specific academic self-concepts in a two-wave longitudinal study. Although Marsh and Yeung found some significant top-down effects, the (horizontal) autoregressive effects were the strongest, and thus, they stated that “the most parsimonious conclusion is that the results support only the horizontal effects” (p. 525).

Regarding across-subject developmental processes, the dimensional comparison theory of academic self-concepts predicts that students compare their individual strengths and weaknesses across different academic subjects (Möller and Marsh, 2013). For example, with such dimensional comparison processes, positive evaluations in one academic subject may yield lower self-evaluations in other subjects (i.e., contrast effect). Such dimensional comparison processes may have important consequences for students' development, namely, that self-concept in one subject will have a negative effect on change in self-concept in other subject (see Parker et al., 2015). For example, the reciprocal internal/external frame of reference model (RI/E model; Möller et al., 2011) predicts small negative effects of academic self-concept on subsequent academic self-concept in noncorresponding subjects (Niepel et al., 2014). To the best of our knowledge, however, this prediction has been tested only a couple of times: Niepel et al. (2014) and Möller et al. (2011) found some support for such negative effects (but see also Parker et al., 2015).

Dimensional comparison theory further predicts that contrast effects across subjects may be smaller or might even become positive (i.e., assimilation effects) when dimensional comparisons are based on domains that are perceived as closely related (Marsh et al., 2014). Empirical studies that have investigated the dimensional comparison processes with subjects other than mathematics and a verbal subject, however, have delivered mixed results. For example, some studies found negative effects between mathematics and science (Chiu, 2012), whereas other found assimilation effects between mathematics and physics (Möller et al., 2006; Jansen et al., 2015). Further, within the verbal domain, some studies found negative effects between two verbal subjects (i.e., Marsh and Yeung, 2001; Marsh et al., 2001; Brunner et al., 2010; Niepel et al., 2014), whereas other studies found no such effects (Xu et al., 2013) or even slightly positive effects (Möller et al., 2006; Marsh et al., 2014).

Notably, previous research on across-subject developmental processes has not taken into account the hierarchical organization of academic self-concepts. Thus, top-down effects of general academic self-concepts on subject-specific academic self-concepts were not controlled for. Consequently, previous estimates of across-subject processes may have confounded developmental processes across subject-specific self-concepts with top-down processes of general academic self-concept. In other words, applying structural models of academic self-concept that take into account the hierarchical organization of the construct can help to disentangle (purely) across-subject processes from top-down processes.

Crucially, top-down, bottom-up, and across-subject developmental processes have never been investigated with regard to academic interest and academic anxiety. However, there is some empirical support from cross-sectional research that dimensional comparison processes can be generalized to academic interest and academic anxiety. Specifically, Schurtz et al. (2014) and Pohlmann (2005) found that achievement affected academic interests in the pattern that is typically found for the dimensional comparison processes. Similarly, Goetz et al. (2008a) found support for dimensional comparison processes with regard to enjoyment (i.e., the emotional component of interest). Likewise, Marsh (1988) found contrast effects with regard to math and English anxieties.

### Longitudinal measurement invariance

Measurement invariance is very important in longitudinal research. Specifically, in order to ensure that the latent constructs have the same substantive meaning over time, invariance in measurement properties is needed so that true changes in the latent constructs can be separated from changes in the operational definitions of the constructs. Crucially, the evaluation of measurement invariance concerns the question of whether or not the manifest indicators are related to their latent factors in the same way at different measurement occasions (Meredith and Horn, 2001). Different degrees of measurement invariance can be differentiated (Meredith, 1993): First, configural invariance requires the number of factors and the pattern of zero and nonzero factor loadings to be equal across time points. Second, metric invariance requires that the corresponding factor loadings are equivalent across time points. When metric invariance has been established, the rank-order stability of latent constructs (McArdle, 2009) as well as the prediction of change can be examined. Analyzing the different degrees of MI can be accomplished by employing longitudinal confirmatory factor models with increasingly more severe restrictions on parameters across time points (Little, 2013).

### Research objectives

The overarching goal of the present study was to examine the developmental dynamics of general and subject-specific (i.e., German, French, and mathematics) components of students' academic self-concept, anxiety, and interest, respectively. Notably, in previous developmental research, the hierarchical relations between general and subject-specific components have rarely been accounted for in a combined model. A vital characteristic of the present study is therefore that we applied longitudinal nested-factor models that capture the hierarchical and subject-specific organization of the each construct. By applying these models, we were able to bring together central lines of research with the aim of making a substantial contribution to a fuller and more nuanced understanding of the developmental dynamics of three key constructs of students' learning-related affect and motivation. First, we analyzed the differential stabilities of the general and subject-specific components. Given that nested-factor models account for the influence of the general construct components on the subject-specific measures, the estimates of the differential stabilities of the subject-specific components of the constructs are not confounded with the stabilities of the general components.

Second, we examined the prediction of change. To this end, we integrated two streams of research that have been separate until now and that have been exemplified for academic self-concepts: the direction of longitudinal causal flow in the construct hierarchy and dimensional comparison effects across different subjects. Notably, given that adequate structural models have not been applied before, these streams have not yet been combined in developmental research on academic self-concepts and have not been addressed at all in (developmental) research on academic anxiety and interest. It is important to note that by bringing these two streams together, we can study how the general components of constructs can affect change in the subject-specific components (top-down processes) and how the subject-specific components can affect change in the general components (bottom-up processes). Moreover, we can also study how subject-specific components can affect change in other subjects (across-subject processes). Given that the general components of the constructs are controlled for in the subject-specific measures, the present analyses can help to disentangle the (pure) across-subject processes occurring between subject-specific components from the top-down effects of the general components on the subject-specific components.

It is important to note that on the basis of the methodological advice given by Cumming (2014) and Bonett (2012) for carrying out replication studies, we conducted our analyses separately on two independent samples with representative longitudinal data from a total of 7361 students attending Grades 7 and 9 in schools in Luxembourg. By doing so, we were able to scrutinize the robustness of the results and to judge the generalizability of our findings.

## Methods

### Samples

The analyses applied in the present study were based on two longitudinal samples of representative data from students who participated in the Luxembourg school-monitoring program (ÉpStan; Martin and Brunner, 2012) at the beginning of the seventh grade as well as at the beginning of the ninth grade. Specifically, Sample 1 (S1) was obtained from the 2010 and 2012 waves and Sample 2 (S2) from the 2011 and 2013 waves of ÉpStan. The main aim of the ÉpStan is to evaluate the key educational outcomes (e.g., subject-specific achievement and students' affective-motivational characteristics) across all state schools in Luxembourg.

From the 4376 students in S1 and 4830 students in S2 who provided data at the first measurement occasion, we excluded students who had more than two missing values on any of the general and subject-specific scales of academic self-concept, interest, and anxiety to ensure valid measurement of the general and subject-specific constructs (*n* = 92 and *n* = 139 for S1 and S2, respectively). Moreover, the analyses in the present study were computed on only the seventh-grade students who also took part in the ninth grade in the respective wave of ÉpStan. As participation in ÉpStan was mandatory for the students, and given the high retention rates in Luxembourgish schools [estimated grade retention rates of 22–24% in lower secondary education, based on data available from both PISA (2009) and Eurostat (2008); see Eurydice, 2011], it is most likely that the students who did not provide data at both waves of measurement consisted primarily of students who repeated a year after the first measurement occasion. A total of 786 students in S1 and 828 students in S2 dropped out. The resulting final sample sizes were thus *N* = 3498 for S1 and *N* = 3863 for S2.

The comparisons of students who provided data at both waves of measurement with students for whom data were available in only the seventh grade revealed that, for both samples, the students who dropped out had significantly lower scores on general academic, mathematics, and German self-concept in the seventh grade (with no significant difference in French self-concept; −0.02 < Cohen's *d* < 0.25). Moreover, these students experienced significantly more anxiety in the school subjects [significant results for the general academic, mathematics, French (only in S2), and German anxiety scales; −0.02 < Cohen's *d* < −0.26]. Regarding interest, they scored significantly lower on the general academic, mathematics, and German interest scales in S1 as well as on the mathematics interest scale in S2 (−0.06 < Cohen's *d* < 0.13). This pattern of results supports the notion that students who provided data only in the seventh grade consisted primarily of students who were held back a grade between the two measurement occasions.

### Measures

The measures of academic self-concept, interest, and anxiety in both longitudinal samples were administered by computer. The instrument consisted of items that covered three core subjects (i.e., mathematics, French, and German) as well as general academic self-concept, general academic interest, and general academic anxiety. Each scale consisted of three items that has undergone extensive pilot testing. In line with other large-scale assessments [e.g., Programme for International Student Assessment (PISA); OECD, 2002, 2005, 2009, 2014], students responded to each item on a rating scale with four categories: disagree, disagree somewhat, agree somewhat, and agree coded as 1, 2, 3, and 4, respectively. All scales showed satisfactory levels of reliability with values for the model-based reliability coefficient ω (see McDonald, 1999; Brunner et al., 2012) ranging from 0.74 to 0.91 in S1 and 0.74 to 0.92 in S2. The wording of the self-concept, anxiety, and interest items is presented in Table A1 in Appendix A of Supplementary Material. Tables A2, A3 in Appendix A of Supplementary Material present descriptive statistics, reliabilities, correlations, and covariances of the scale scores that were obtained for both longitudinal samples, S1 and S2, respectively.

#### Academic self-concept measures

The academic self-concept instruments consisted of items taken from the Self-Description Questionnaire (SDQ; e.g., Marsh and O'Neill, 1984), which is considered to be one of the best self-concept instruments available (e.g., Byrne, 1996), and were adapted to the respective subjects according to the instructions provided by Marsh (1990).

#### Academic interest measures

The academic interest instruments consisted of items that were developed according to the corresponding construct definitions (Schiefele, 1991; Renninger, 2000; Krapp, 2002); that is, one item assessed feelings of personal importance and one item emotional value. In addition, one global item was constructed with the aim of directly and maximally representing the essence of the definition of academic interest (e.g., “I am interested in French” for the subject of French or “I am interested in most school subjects” for the general level).

#### Academic anxiety measures

The academic anxiety instruments consisted of items that were developed according to the corresponding construct definitions (Liebert and Morris, 1967; Zeidner, 2007); that is, one item assessed the worry component and one the emotionality component of academic anxiety. In addition, one global item was constructed with the aim of directly and maximally representing the essence of the definition of academic anxiety (e.g., “I am afraid of most school subjects”).

### Statistical analyses

#### Model specification

**Figure 2:**
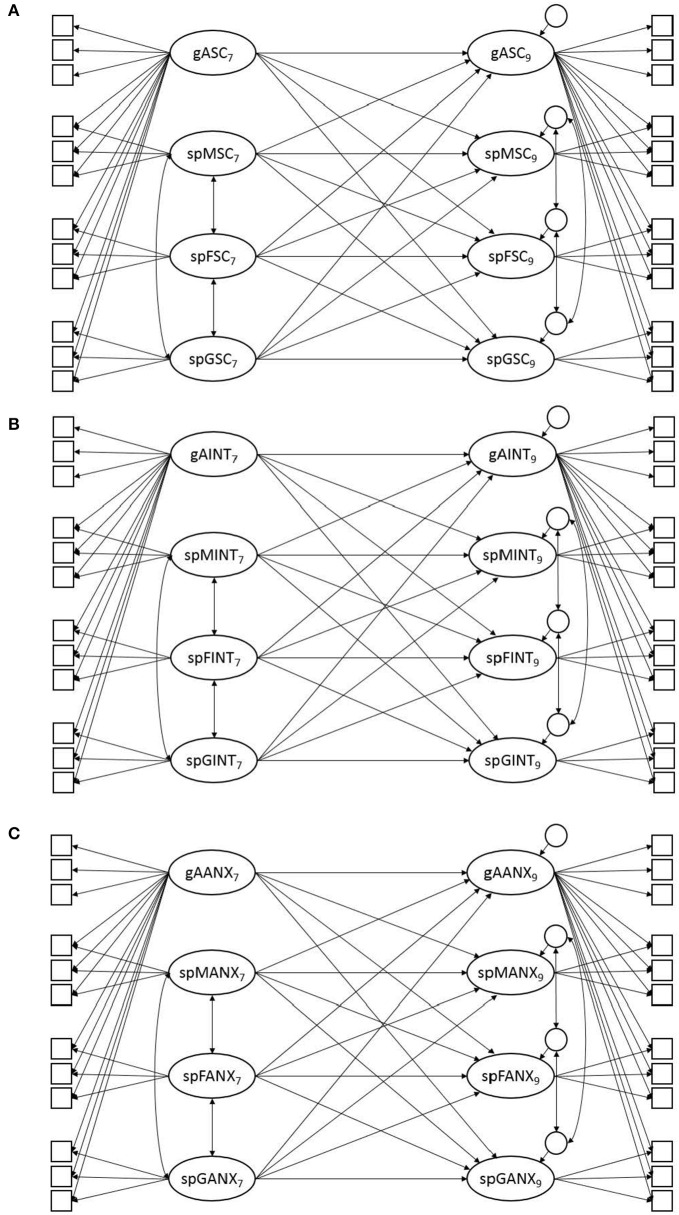
**Schematic diagrams of the longitudinal nested-factor models for (A) academic self-concept, (B) academic interest, and (C) academic anxiety**. Residuals and their across-time correlations are not depicted in the model to ensure the clarity of the figure. The suffixes 7 and 9 in the factor names indicate Grades 7 and 9, respectively. gASC = general academic self-concept; spMSC = specific mathematics self-concept; spFSC = specific French self-concept; spGSC = specific German self-concept; gAINT = general academic interest; spMINT = specific mathematics interest; spFINT = specific French interest; spGINT = specific German interest; gAANX = general academic anxiety; spMANX = specific mathematics anxiety; spFANX = specific French anxiety; spGANX = specific German anxiety.

Longitudinal confirmatory factor analyses were used to assess measurement invariance as well as the differential stability and prediction of change in the affective-motivational constructs. Specifically, in the longitudinal nested-factor models for academic self-concept, academic interest, and academic anxiety, the general factors (i.e., gASC, gINT, gANX) and the subject-specific factors (e.g., spMSC, spFSC, spGSC) were specified to be correlated with or regressed on each other across time (see Figure 2). All statistical analyses were computed separately for each sample so that the robustness of the results could be scrutinized.

The latent variables were measured with the items (as described above) that reflected the corresponding general or subject-specific constructs. In the configural invariance models (i.e., AS.1, AI.1, and AA.1), the latent variables were identified by fixing their variance to 1. The factor loadings and residual variances were freely estimated. Furthermore, we set the means of all latent factors to zero and freely estimated the intercepts of the manifest indicators. To test for metric invariance (i.e., Models AS.2, AI.2, and AA.2), the variance of the factors at the first measurement occasion (Grade 7) were fixed to 1, whereas for the other measurement occasion (Grade 9), the factor variances were freely estimated (Bontempo et al., 2012).

The residual terms for the general and subject-specific items may capture both indicator-specific variance and random measurement error. In longitudinal studies, the residual terms are therefore often correlated over time to account for reliable indicator-specific variance in the residual terms (Cole and Maxwell, 2003; Little, 2013) because not accounting for these correlations can lead to misfit and bias in parameter estimates (e.g., the overestimation of stability coefficients; Geiser et al., 2010). Therefore, we allowed the residual terms of all corresponding items to be correlated across time. Moreover, correlations between the residual terms of items with parallel wording were included in the models to obtain accurate parameter estimates (Marsh et al., 1997).

#### Missing data and the nested data structure

Missing data are unavoidable in any large-scale assessment. The highest sample-specific percentages of missing data in the final sample were 1.9% in S1 for item AX_A1_7, which assessed general academic anxiety in the seventh grade, and 1.5% in S2 for item SC_M3_7, which assessed self-concept in mathematics in the seventh grade. We used the full information maximum likelihood procedure (FIML) implemented in Mplus to account for the pattern of missing data as observed in the present study. Moreover, the “complex” option in Mplus (with class in the seventh grade as a cluster variable) was used to obtain standard errors and fit statistics that were corrected for the nonindependence of observations given that the students were not independently sampled but rather nested within classes. The model parameters were estimated by the MLR estimator, which is an appropriate variant of the maximum likelihood estimator (ML) for data with missing values and nonindependence of observations (see Muthén and Muthén, 1998–2012).

#### Examining measurement invariance

We tested the measurement invariance of the investigated models in two consecutive steps. In the first step, we tested for configural invariance (i.e., AS.1, AI.1, and AA.1), which requires the same pattern of zero and nonzero factor loadings across the time points. Second, we additionally constrained the unstandardized factor loadings of corresponding items to be equal across time to test for metric invariance (i.e., AS.2, AI.2, and AA.2). To evaluate measurement invariance, first, we examined the fit of the models by computing a chi-square test of overall model fit as well as the recommended descriptive fit indices. Second, when the overall model fit was satisfactory, we examined the difference in model fit between the less and the more constrained models (see Appendix B in Supplementary Materials for details).

#### Examining differential stabilities

After establishing measurement invariance, we examined the differential stability of the general and subject-specific components of academic self-concept, interest, and anxiety. To this end, we analyzed the autocorrelations of the corresponding latent variables across time.

#### Examining the causes of change

Subsequently, we regressed the latent variables that represented the general or subject-specific components of the constructs in Grade 9 on the latent variables representing the general or subject-specific components in Grade 7. Specifically, as the directed paths that link the corresponding factors between the time points are called autoregressions and account for individual differences stability of the factors across time, the directed paths from other factors indicate influences that are predictive of the cross-time changes (see Little et al., 2006). These effects can thus indicate whether interindividual differences in change in general or subject-specific components in Grade 9 are related to prior status in general or subject-specific components in Grade 7.

## Results

### Measurement invariance

The results of the analyses of measurement invariance can be summarized as follows (see Appendix B in Supplementary Materials for a detailed description of these analyses and the model fit results for the invariance conditions specified in Table B1 in Appendix B of Supplementary Materials): For all constructs in both samples, the models specifying configural and metric invariance provided an adequate overall fit to the data, and the differences in model fit between the less and the more constrained models were acceptable. The adequate fit of the metric-invariant model specifications indicated that the nested-factor models were appropriate for representing the structural relations of the general and subject-specific components of the respective construct in both grade levels and that the corresponding latent variables had the same meaning across time.

Moreover, the factor loadings on all factors were substantial in both samples, showing that the latent variables representing general academic self-concept, interest, and anxiety as well as the subject-specific factors were well-defined for students in Grades 7 and 9 (see Tables B2–B4 in Appendix B of Supplementary Materials for the complete factor loading matrices obtained for the constrained models, AS.2, AI.2, and AA.2, respectively). This pattern of results supported the hierarchical and multidimensional structure of the constructs. Finally, the subject-specific factors were negatively related across the different subjects in all models and at both time points (see Tables B2–B4 in Appendix B of Supplementary Materials for the correlations between factors obtained for the academic self-concept, interest, and anxiety models, respectively). This result indicates separation between the subject-specific components for all constructs.

### Differential stabilities

The confirmation of metric invariance for the nested-factor models of academic self-concept, interest, and anxiety indicated that further analyses on the developmental dynamics of these constructs could be justified. Differential stabilities for the general components of the constructs were highly consistent across samples. Values ranged between *r* = 0.42 for academic self-concept and *r* = 0.48 for academic anxiety in S1 and between *r* = 0.42 for academic interest and *r* = 0.55 for academic anxiety in S2 (see Table 1). The differential stability coefficients for the subject-specific components were also highly consistent across samples: For academic self-concept, the values ranged from *r* = 0.56/0.54 (mathematics; in S1/S2) to *r* = 0.73/0.72 (French). Autocorrelations for the subject-specific interest components ranged from *r* = 0.47/0.45 (mathematics) to *r* = 0.61/0.57 (French). Differential stability for the subject-specific anxiety components ranged from *r* = 0.45/0.48 (mathematics) to *r* = 0.60/0.57 (German). Overall, the autocorrelations observed for the general and subject-specific components of the academic self-concept, interest, and anxiety factors were positive and indicated moderate levels of differential stability, respectively.

**Table 1:** **Correlations between general and subject-specific components over time and their 95% Confidence Intervals [CIs] obtained for the academic self-concept, academic interest, and academic anxiety models**.

	**Sample 1**				**Sample 2**			
**ACADEMIC SELF-CONCEPT**								
	gASC_9_	spMSC_9_	spFSC_9_	spGSC_9_	gASC_9_	spMSC_9_	spFSC_9_	spGSC_9_
gASC_7_	**0.42**	0.01	−0.05	0.05	**0.44**	0.04	−0.06	0.03
	**[0.38, 0.47]**	[−0.03, 0.05]	[−0.09, −0.02]	[0.01, 0.09]	**[0.40, 0.48]**	[0.00, 0.07]	[−0.09, −0.03]	[0.00, 0.07]
spMSC_7_	−0.01	**0.56**	−0.19	−0.15	0.03	**0.54**	−0.14	−0.21
	[−0.05, 0.02]	**[0.52, 0.60]**	[−0.23, −0.02]	[−0.19, 0.09]	[−0.01, 0.07]	**[0.50, 0.57]**	[−0.18, −0.10]	[−0.25, −0.17]
spFSC_7_	0.01	−0.14	**0.73**	−0.47	0.01	−0.17	**0.72**	−0.45
	[−0.03, 0.04]	[−0.18, −0.10]	**[0.71**, −**0.02]**	[−0.51, 0.09]	[−0.02, 0.04]	[−0.21, −0.14]	**[0.70, 0.74]**	[−0.48, −0.41]
spGSC_7_	0.04	−0.23	−0.47	**0.64**	0.01	−0.21	−0.46	**0.65**
	[−0.01, 0.07]	[−0.28, −0.19]	[−0.50, −0.02]	**[0.61, 0.09]**	[−0.02, 0.04]	[−0.25, −0.17]	[−0.49, −0.43]	**[0.62, 0.68]**
**ACADEMIC INTEREST**								
	gAINT_9_	spMINT_9_	spFINT_9_	spGINT_9_	gAINT_9_	spMINT_9_	spFINT_9_	spGINT_9_
gAINT_7_	**0.46**	0.03	0.00	0.03	**0.42**	−0.01	0.03	0.02
	**[0.42, 0.5]**	[−0.01, 0.07]	[−0.04, 0.04]	[−0.01, 0.07]	**[0.38, 0.46]**	[−0.05, 0.03]	[−0.01, 0.06]	[−0.02, 0.06]
spMINT_7_	−0.02	**0.47**	−0.10	−0.07	0.01	**0.45**	−0.09	−0.15
	[−0.06, 0.02]	**[0.43, 0.51]**	[−0.14, −0.06]	[−0.11, −0.02]	[−0.03, 0.05]	**[0.41, 0.48]**	[−0.13, −0.04]	[−0.19, −0.11]
spFINT_7_	0.04	−0.09	**0.61**	−0.31	0.06	−0.12	**0.57**	−0.28
	[0.00, 0.08]	[−0.13, −0.04]	**[0.57, 0.64]**	[−0.36, −0.27]	[0.02, 0.10]	[−0.16, −0.08]	**[0.53, 0.61]**	[−0.32, −0.24]
spGINT_7_	0.01	−0.15	−0.35	**0.48**	−0.01	−0.10	−0.31	**0.49**
	[−0.03, 0.05]	[−0.19, −0.11]	[−0.39, −0.30]	**[0.44, 0.52]**	[−0.05, 0.03]	[−0.14, −0.07]	[−0.35, −0.26]	**[0.45, 0.53]**
**ACADEMIC ANXIETY**								
	gAANX_9_	spMANX_9_	spFANX_9_	spGANX_9_	gAANX_9_	spMANX_9_	spFANX_9_	spGANX_9_
gAANX_7_	**0.48**	0.02	−0.06	0.01	**0.55**	0.03	−0.06	0.00
	**[0.44, 0.52]**	[−0.02, 0.06]	[−0.10, −0.02]	[−0.04, 0.05]	**[0.52, 0.58]**	[−0.01, 0.07]	[−0.09, −0.02]	[−0.04, 0.04]
spMANX_7_	0.02	**0.45**	−0.14	−0.19	0.01	**0.48**	−0.09	−0.19
	[−0.03, 0.07]	**[0.38, 0.51]**	[−0.20, −0.08]	[−0.26, −0.12]	[−0.03, 0.06]	**[0.43, 0.52]**	[−0.14, −0.04]	[−0.24, −0.13]
spFANX_7_	−0.03	−0.12	**0.50**	−0.32	−0.04	−0.14	**0.51**	−0.29
	[−0.07, 0.01]	[−0.18, −0.06]	**[0.45, 0.55]**	[−0.38, −0.26]	[−0.08, −0.01]	[−0.19, −0.08]	**[0.47, 0.56]**	[−0.34, −0.24]
spGANX_7_	0.05	−0.21	−0.29	**0.60**	0.03	−0.17	−0.29	**0.57**
	[0.00, 0.09]	[−0.27, −0.15]	[−0.34, −0.24]	**[0.54, 0.65]**	[−0.01, 0.07]	[−0.23, −0.12]	[−0.34, −0.24]	**[0.52, 0.62]**

gASC, general academic self-concept; spMSC, specific mathematics self-concept; spFSC, specific French self-concept; spGSC, specific German self-concept; gAINT, general academic interest; spMINT, specific mathematics interest; spFINT, specific French interest; spGINT, specific German interest; gAANX, general academic anxiety; spMANX, specific mathematics anxiety; spFANX, specific French anxiety; spGANX, specific German anxiety; The suffixes 7 and 9 in the factor names indicate Grades 7 and 9, respectively. Autocorrelations of factors (i.e., stability coefficients) are in bold.

### Prediction of change

The autocorrelations obtained for the general and subject-specific components of the constructs were uncontaminated by measurement error, and thus, autocorrelations less than 1 could be interpreted as indicative of interindividual differences in intraindividual change (Nesselroade, 1991). All autocorrelations depicted in Table 1 as well as all autoregressions depicted in Table 2 were clearly less than 1, thus implying that there were substantial reliable individual differences in change in the general and subject-specific components of academic self-concept, anxiety, and interest, respectively. How can these interindividual differences in change be explained?

**Table 2:** **Standardized regression coefficients between general and subject-specific components over time and their 95% Confidence Intervals [CIs] obtained for the academic self–concept, academic interest, and academic anxiety models**.

	**Sample 1**				**Sample 2**			
**ACADEMIC SELF-CONCEPT**								
	gASC_9_	spMSC_9_	spFSC_9_	spGSC_9_	gASC_9_	spMSC_9_	spFSC_9_	spGSC_9_
gASC_7_	0.42	0.01	−0.05	0.05	0.44	0.04	−0.06	0.03
	[0.38, 0.47]	[−0.03, 0.05]	[−0.09, −0.02]	[0.01, 0.09]	[0.40, 0.48]	[0.00, 0.07]	[−0.09, −0.03]	[0.00, 0.07]
spMSC_7_	0.02	0.49	−0.07	−0.07	0.06	0.46	−0.04	−0.12
	[−0.04, 0.07]	[0.44, 0.54]	[−0.12, −0.03]	[−0.12, −0.02]	[0.00, 0.11]	[0.42, 0.51]	[−0.08, 0]	[−0.16, −0.07]
spFSC_7_	0.05	−0.11	0.63	−0.21	0.05	−0.14	0.64	−0.22
	[−0.02, 0.11]	[−0.17, −0.05]	[0.59, 0.68]	[−0.27, −0.15]	[−0.01, 0.11]	[−0.19, −0.09]	[0.59, 0.68]	[−0.27, −0.16]
spGSC_7_	0.07	−0.17	−0.15	0.51	0.05	−0.15	−0.15	0.51
	[−0.01, 0.14]	[−0.23, −0.10]	[−0.20, −0.10]	[0.45, 0.57]	[−0.01, 0.11]	[−0.20, −0.10]	[−0.20, −0.11]	[0.46, 0.56]
**ACADEMIC INTEREST**								
	gAINT_9_	spMINT_9_	spFINT_9_	spGINT_9_	gAINT_9_	spMINT_9_	spFINT_9_	spGINT_9_
gAINT_7_	0.46	0.03	0.00	0.03	0.42	−0.01	0.03	0.02
	[0.42, 0.5]	[−0.01, 0.07]	[−0.04, 0.04]	[−0.01, 0.07]	[0.38, 0.46]	[−0.05, 0.03]	[−0.01, 0.06]	[−0.02, 0.06]
spMINT_7_	−0.01	0.45	−0.05	−0.05	0.02	0.43	−0.05	−0.12
	[−0.06, 0.03]	[0.41, 0.49]	[−0.09, −0.01]	[−0.10, 0.00]	[−0.03, 0.06]	[0.39, 0.46]	[−0.09, −0.01]	[−0.16, −0.08]
spFINT_7_	0.05	−0.06	0.54	−0.19	0.07	−0.10	0.52	−0.18
	[0.00, 0.10]	[−0.11, −0.01]	[0.50, 0.58]	[−0.23, −0.14]	[0.02, 0.11]	[−0.14, −0.06]	[0.47, 0.56]	[−0.22, −0.13]
spGINT_7_	0.03	−0.12	−0.18	0.42	0.01	−0.08	−0.16	0.42
	[−0.02, 0.07]	[−0.16, −0.07]	[−0.22, −0.14]	[0.37, 0.46]	[−0.03, 0.05]	[−0.12, −0.04]	[−0.21, −0.12]	[0.38, 0.46]
**ACADEMIC ANXIETY**								
	gAANX_9_	spMANX_9_	spFANX_9_	spGANX_9_	gAANX_9_	spMANX_9_	spFANX_9_	spGANX_9_
gAANX_7_	0.48	0.02	−0.06	0.01	0.55	0.03	−0.06	0.00
	[0.44, 0.52]	[−0.02, 0.06]	[−0.10, −0.02]	[−0.04, 0.05]	[0.52, 0.58]	[−0.01, 0.07]	[−0.09, −0.02]	[−0.04, 0.04]
spMANX_7_	0.04	0.40	−0.09	−0.05	0.02	0.45	−0.05	0.00
	[−0.04, 0.12]	[0.30, 0.50]	[−0.18, 0.01]	[−0.15, 0.05]	[−0.05, 0.09]	[0.37, 0.52]	[−0.12, 0.03]	[−0.09, 0.08]
spFANX_7_	0.00	−0.06	0.42	−0.12	−0.03	−0.08	0.46	−0.06
	[−0.07, 0.08]	[−0.17, 0.04]	[0.33, 0.50]	[−0.22, −0.02]	[−0.10, 0.04]	[−0.16, 0.01]	[0.38, 0.53]	[−0.14, 0.02]
spGANX_7_	0.06	−0.11	−0.15	0.54	0.03	−0.04	−0.11	0.55
	[−0.02, 0.15]	[−0.21, 0.00]	[−0.24, −0.06]	[0.43, 0.64]	[−0.05, 0.10]	[−0.13, 0.05]	[−0.20, −0.03]	[0.45, 0.64]

gASC, general academic self-concept; spMSC, specific mathematics self-concept; spFSC, specific French self-concept; spGSC, specific German self-concept; gAINT,general academic interest; spMINT, specific mathematics interest; spFINT, specific French interest; spGINT, specific German interest; gAANX, general academic anxiety; spMANX, specific mathematics anxiety; spFANX, specific French anxiety; spGANX, specific German anxiety; The suffixes 7 and 9 in the factor names indicate Grades 7 and 9, respectively.

First, we found little evidence for substantial top-down processes in which the general components affected change in the subject-specific components of the constructs. The values for the corresponding standardized regression coefficients β were negligible for academic self-concept (−0.05 ≤ β ≤ 0.05 in S1; −0.06 ≤ β ≤ 0.04 in S2), interest (0.00 ≤ β ≤ 0.03 in S1; −0.01 ≤ β ≤ 0.03 in S2), and anxiety (−0.06 ≤ β ≤ 0.02 in S1; −0.06 ≤ β ≤ 0.03 in S2).

Second, we also found little evidence for substantial bottom-up processes in which the subject-specific components affected change in the general components of the constructs. The values for the corresponding standardized regression coefficients were negligible for academic self-concept (0.02 ≤ β ≤ 0.07/0.05 ≤ β ≤ 0.06), interest (−0.01 ≤ β ≤ 0.05/0.01 ≤ β ≤ 0.07), and anxiety (0.00 ≤ β ≤ 0.06/-0.03 ≤ β ≤ 0.03) in S1 and S2, respectively.

Third, we found evidence for substantial (i.e., ∣ β ∣ ≥ 0.10) negative effects of subject-specific components in Grade 7 on change in the specific components of other subjects in Grade 9. These effects can be interpreted as (negative) across-subject comparison processes.

As for academic self-concept, specific French self-concept in Grade 7 was negatively related to change in specific German self-concept in Grade 9 in both samples (β = −0.21/−0.22); specific German self-concept in Grade 7, on the other hand, was negatively related to change in specific French self-concept 2 years later in both samples (β = −0.15/−0.15). Further, we found that change in specific math self-concept in Grade 9 was consistently negatively related to specific German self-concept (β = −0.17/−0.15) and specific French self-concept (β = −0.11/−0.14) in Grade 7 in both samples. Finally, albeit negative, specific math self-concept in Grade 7 showed no substantial relations to change in specific German self-concept or change in specific French self-concept in Grade 9 (with all ∣ βs ∣ < 0.10) with one exception (i.e., spGSC9 regressed on spMSC7 with β = −0.12 in S2).

It is important to note that the patterns of results obtained for interest and academic anxiety demonstrated several similarities but also some differences compared with the pattern observed for academic self-concept. Particularly, in both samples, specific interest in French in Grade 7 was negatively related to change in specific interest in German in Grade 9 (β = −0.19/−0.18); specific interest in German in Grade 7, on the other hand, was negatively related to change in specific interest in French self−concept 2 years later (β = −0.18/−0.16). The relation of change in specific interest in mathematics in Grade 9 to specific interest in German in Grade 7 was substantial in S1 (β = −0.12) but not in S2 (β = −0.08); its relation to specific interest in French in Grade 7 was substantial in S2 (β = −0.10) but not in S1 (β = −0.06). Finally, albeit negative, specific interest in mathematics in Grade 7 showed no substantial relations to change in specific interest in German and change in specific interest in French in Grade 9 (with ∣ βs ∣ < 0.10), with only one exception (i.e., spGINT9 regressed on spMINT7 with β = −0.12 in S2).

Finally, as for academic anxiety, specific anxiety in French in Grade 7 was negatively related to change in specific anxiety in German in Grade 9 in S1 (β = −0.12) but not in S2 (β = −0.06); specific anxiety in German in Grade 7, on the other hand, was negatively related to change in specific anxiety in French 2 years later in both samples (β = −0.15/−0.11). The relation of change in specific anxiety in mathematics in Grade 9 to specific anxiety in German in Grade 7 was substantial in S1 (β = −0.11) but not in S2 (β = −0.04); its relation to specific anxiety in French in Grade 7 was not substantial in any sample (β = −0.06/−0.08). Finally, specific anxiety in mathematics in Grade 7 was not substantially related to change in specific anxiety in German or change in specific anxiety in French in Grade 9 (with ∣ βs ∣ < 0.10).

## Discussion

The overarching goal of the present study was to examine the developmental dynamics of the general and subject-specific components of students' academic self-concept, anxiety, and interest, respectively. Following the methodological advice given by Cumming (2014) and Bonett (2012) for carrying out replication studies, we drew on two representative longitudinal samples to tackle two key objectives of developmental research (Baltes and Nesselroade, 1979): to analyze differential stabilities and to predict change in these affective-motivational constructs. In doing so, the present results empirically underscore several vital structural and developmental characteristics that are shared by academic self-concept, academic interest, and academic anxiety. Our discussion of the major findings of the present study will focus on the results that were replicated in both samples as these findings demonstrate broad generalizability and robustness.

First, the multidimensional and hierarchical organization of self-concept with general academic self-concept operating at the apex of the hierarchy has received ample empirical support by research on the nested Marsh/Shavelson model (Brunner et al., 2008, 2009, 2010). However, regarding academic interest and academic anxiety, although general and subject-specific conceptualizations appear to coexist in the literature, previous research did not formally relate the two approaches to each other: Some scholars have conceived of academic interest as strongly subject-specific (e.g., Schiefele, 1991; Krapp, 2002; Hidi and Renninger, 2006), whereas another emphasized the idea that students may also have a general individual interest in learning (Ainley et al., 2002). Likewise, in the last 10–15 years, academic anxiety has been considered to be specific to subjects (Goetz et al., 2007), whereas earlier research focused on the general nature of academic anxiety (Zeidner, 1998). Therefore, a vital strength of the present study was that we applied nested-factor models that captured the subject-specific nature of these constructs as well as the hierarchical relations between the general and subject-specific components of the constructs to integrate subject-specific and general approaches of academic interest and academic anxiety (and, of course, academic self-concept). By doing so, we were able to show that academic self-concept, interest, and anxiety share (at least for students in Grades 7 and 9) vital structural characteristics: (a) a multidimensional nature with respect to different subjects, (b) a hierarchical organization with a general component at the apex of the hierarchy, and (c) a strong separation between the subject-specific components.

Second, the nested-factor models accounted for the influence of the general components of the constructs on the subject-specific measures. This has important advantages when studying individual development, for example, because differential stabilities of subject-specific construct components are not confounded with the stabilities of general construct components. Regarding the differential stabilities of general as well as subject-specific components of academic self-concept, interest, and anxiety, moderate levels of stability were observed. Thus, there is a substantial level of differential stability in the individual configuration of the general level as well as the subject-specific strengths and weaknesses of students' profiles of these affective-motivational constructs. The highest stability coefficients were observed for self-concept in French and German. This result is somewhat contrary to the seminal theoretical conceptualization of self-concept by Shavelson et al. (1976), where general academic self-concept was predicted to show higher levels of differential stability than the subject-specific self-concepts. Moreover, it is interesting that the lowest stabilities of subject-specific affective-motivational components were observed for mathematics. This result may be associated with the significance that this subject gains as students advance in their school careers. For many students, mathematics is a domain that gains (or loses) substantive importance, and this in turn may affect the differential stability of students' self-concept, interest, and anxiety in mathematics.

Third, theoretical considerations of the direction of causal flow in the self-concept hierarchy have been ambiguous. Several scholars have predicted bottom-up processes that flow from subject-specific to general academic self-concept (e.g., Shavelson et al., 1976; Rosenberg, 1979; Harter, 1986). Brown (1993), on the other hand, argued for top-down processes that flow from general to subject-specific self-concepts. Given that structural models that integrate hierarchical relations between general and subject-specific components have not been tested for academic interest or anxiety, this question has not been empirically addressed before for these two constructs. However, but well in line with the results of Marsh and Yeung (1998) still unique study of academic self-concept, we did not find support for longitudinal (a) top-down or (b) bottom-up processes that affect change in subject-specific or general components for any of the constructs under investigation.

Fourth, our results showed that change in the subject-specific components of academic self-concept, interest, and anxiety could be partially explained by negative effects between noncorresponding subjects. These results are in line with predictions from the RI/E model and the dimensional comparison theory (Möller and Marsh, 2013). These theories imply that self-concept in one subject has a negative effect on change in other domains (see Parker et al., 2015) especially when the subjects are not closely related (Marsh et al., 2015). Given that the general components of the constructs were controlled for in the subject-specific measures, the present analyses strongly support the idea that these across-subject processes are (a) operating at the level of subjects and (b) do not result from (additional) top-down processes that flow from the general components to the subject-specific components.

Moreover, the present study extends findings on across-subject developmental processes to the realm of academic interest and anxiety. Specifically, we found substantial developmental processes between specific French and specific German components for all constructs that we investigated. Thus, these findings are well aligned with results from other studies that have investigated self-concept formation for languages that are vital in students' lives (Marsh and Yeung, 2001; Marsh et al., 2001; Brunner et al., 2010). The developmental effects between mathematics and the two specific verbal components were in most cases slightly lower yet in many cases still substantial. It is important to note that these results (in combination with the moderate stabilities of the general and subject-specific components of the constructs) indicate that students' affective-motivational profile shapes become magnified over time. This means that profile differences between verbal subjects (i.e., French and German) become larger but so do differences between mathematics on the one side and verbal subjects on the other. For example, a student who has a strong self-concept in German tends to (a) retain his or her level of German self-concept, (b) develop a weaker self-concept in French, and (c) develop a weaker self-concept in mathematics as well. As affective-motivational constructs determine academic effort, choices, and success, these (across-subject) developmental processes may have important implications for students' future educational careers (e.g., as students tend to select courses and curricula that match their affective-motivational profiles).

### Limitations and outlook

Certain limitations should be considered when interpreting the results of our study. First, the generalizability of our results may be limited by the fact that the data were obtained only from samples of adolescents in Luxembourg. For example, there are indications that the relations between the language-specific self-concepts may depend on the role of the languages in the various curricula and societies (see Brunner et al., 2010). In Luxembourg, both German and French play important roles in school and society. Therefore, further research is needed to investigate whether the across-subject processes found in the present study can also be found in different cultural contexts.

Second, we capitalized on data from two representative samples with students attending Grades 7 and 9. Future research may benefit from collecting data with a larger number of measurement points (e.g., in Grade 8) to allow for analyses with a higher resolution of the developmental processes that affect stability and change in students' affective-motivational constructs. Specifically, stronger relations may be expected for general but also subject-specific construct components when the time lags are shorter. Thus, the results in the present study (with a time lag of 2 years) can be interpreted as lower-bound estimates. Further, by focusing on substantial coefficients (i.e., ∣ βs ∣ ≥ 0.10) and on the results that were found in both samples, we might have missed some dynamics that have smaller effects on students' affective and motivational development.

Next, the stability of affective-motivational constructs were examined only with regard to change from the seventh to the ninth grade. Stability across a broader time frame should be investigated to obtain a fuller understanding. Specifically, as students in Luxembourg are assigned to different secondary tracks at the end of Grade 6 and therefore have to accommodate to new social frames so that external and internal comparisons become actualized, the stability coefficients and cross-lagged effects found in the present study may be viewed as lower bound estimates.

Third, in nested-factor models, the subject-specific components are uncorrelated with the general components. However, this assumption may be unrealistic in some situations (Marsh and Grayson, 1995; Pohl et al., 2008). In fact, it might be quite reasonable from a substantial point of view to allow for correlations between these components. It might, for example, be possible that the subject-specific deviations from the general level of affective-motivational constructs covary with the general level of the constructs. Hence, future research may benefit from studying development in students' affective-motivational constructs by using, for example, the latent difference model, which allows for such correlations (Pohl et al., 2008; Geiser et al., 2012).

### Implications

Regarding implications for future research, our results show that the different affective-motivational constructs share vital characteristics concerning (a) their structure and (b) their developmental dynamics. Therefore, a comprehensive longitudinal structural model, analogous to Gogol et al.'s (submitted) integrative model, could be developed to parsimoniously integrate the structural and developmental similarities of different affective-motivational constructs in a single model. Moreover, negative comparison processes across subjects imply that several subject-specific components have a negative effect on change in specific components of other subjects. These effects suggest that intervention efforts intended to increase academic self-concept or interests and to decrease students' academic anxiety should not rely on targeting one subject alone but rather should take into consideration students' subject-specific affective-motivational experiences in other subjects (see also Parker et al., 2015).

## Author contributions

This work exploited existing data sets. The corresponding data collections were authorized by the Ministry of Education and the ethical issues were thus fully covered; the Luxembourg Commission nationale pour la protection des données (National Commission for Data Protection; CNPD) had been notified by the Ministry of Education about the conduct of the studies.

## Author contributions

KG, MB, FP, TG, and RM substantially contributed to the conception of the work, interpretation of data for the work, revising the work critically for important intellectual content as well as approved the final version of the work to be published and agree to be accountable for all aspects of the work in ensuring that questions related to the accuracy or integrity of any part of the work are appropriately investigated and resolved. KG analyzed the data. KG and MB substantially contributed to drafting the work.

## Funding

This work was supported by the Fonds National de la Recherche (FNR) of Luxembourg (AFR grant). The funder had no role in study design, data collection and analysis, decision to publish, or preparation of the manuscript.

### Conflict of interest statement

The authors declare that the research was conducted in the absence of any commercial or financial relationships that could be construed as a potential conflict of interest.
