# Supplementary material for: Developmental Dynamics of General and School-Subject-Specific Components of Academic Self-Concept, Academic Interest, and Academic Anxiety
Source: Front Psychol. 2016 Mar 17;7:356. doi: 10.3389/fpsyg.2016.00356 (PMC4794478; doi:10.3389/fpsyg.2016.00356)
Supplement: Supplementary file 1 [file AppendixB.DOCX]

Supplementary Material

Developmental Dynamics of General and School-Subject-Specific Components of Academic Self-Concept, Academic Interest, and Academic Anxiety

**Katarzyna Gogol*, Martin Brunner, Franzis Preckel, Thomas Goetz, Romain Martin**

*** Correspondence:** Katarzyna Gogol: katarzyna.gogol@fu-berlin.de

# Supplemental appendix B: Measurement invariance

## Examining measurement invariance

To evaluate measurement invariance of the investigated models, first, we examined the fit of the models by computing a chi-square test of the overall model fit as well as the recommended descriptive fit indices (Hu & Bentler, 1998) such as the Standardized Root Mean Square Residual (SRMR), the Comparative Fit Index (CFI), and the Root Mean Square Error of Approximation (RMSEA). The CFI values were calculated with respect to a null model (i.e., Model 0A) that is appropriate for investigating measurement invariance as suggested by Widaman and Thompson (2003): In addition to being specified as mutually uncorrelated, the variances and means of the manifest variables were constrained to be equal for the seventh and ninth grades. SRMR values below .08, RMSEA values below .06, and CFI values greater than .95 are usually considered to reflect good model fit (Browne & Cudeck, 1993; Hu & Bentler, 1998). Second, when the overall model fit was satisfactory, we examined the difference in model fit between the less and the more constrained models. Specifically, we inspected differences in the χ2 goodness-of-fit statistic (∆χ2) and differences in the descriptive fit indices (∆RMSEA, ∆SRMR, and ∆CFI). According to Chen (2007) and Cheung and Rensvold (2002), a change in fit of less than or equal to .01 for the CFI as well as less than .015 for the RMSEA can be treated as support for the more constrained model. In a more recent study, Meade, Johnson, and Braddy (2008) recommended a more conservative cut-off value of .002 for the decrease in the CFI. However, it has to be noted that these recommendations were formulated on the basis of simple-structure models (each indicator loads on only one factor). Khojasteh and Lo (2015) recommended a cut-off of .003 to .004 for the change in the CFI for evaluating metric invariance in nested-factor models in which the indicators load on two factors.

## Results for measurement invariance

Model fit statistics for the specified invariance conditions are reported in Table B1. The p-values for the χ2 statistics for all models in both samples were below .01, indicating statistically significant discrepancies between the hypothesized model and the observed data. However, it is well-known that the χ2 statistic is sensitive to sample size, whereby trivial model misfit may result in significant values with modest sample sizes (Iacobucci, 2010). Given the large sample sizes in the present study, we therefore focused our evaluation of model fit on the descriptive fit indices. The descriptive fit indices indicated that for all constructs and in both samples, the specified nested-factor models with configural as well as metric invariance provided an adequate overall fit to the data according to the recommended benchmark values. Further, the χ2 goodness-of-fit statistic deteriorated significantly (at p < .01) in both samples with the factor loadings constraint that was introduced. However, the χ2 difference test is considered to be too sensitive to trivial fluctuations and differences in the context of invariance testing (Little, 2013, p. 155), especially with large samples. Notably, with respect to descriptive model fit indices, the differences in model fit between the less and the more constrained models were acceptable for all constructs in both samples, even when the more conservative cut-off values were applied.

| Table B1 | | | | | | | | | | | |
| --- | --- | --- | --- | --- | --- | --- | --- | --- | --- | --- | --- |
| *Investigation of Measurement Invariance: Fit Statistics Obtained for the Nested-Factor Model* | | | | | | | | | | | |
| Model | χ^2^ | *df* | CFI | RMSEA | SRMR | Model comparison | | | | | |
|  |  |  |  |  |  | Compare | *∆*χ^2^ | *∆df* | *∆*CFI | *∆*RMSEA | *∆*SRMR |
| *Sample 1* |  |  |  |  |  |  |  |  |  |  |  |
| AS.1 | 565.81 | 170 | .991 | .026 | .019 |  |  |  |  |  |  |
| AS.2 | 610.04 | 187 | .990 | .025 | .020 | AS.2 vs. AS.1 | 44.23 | 17 | -.001 | -.001 | .001 |
| AI.1 | 629.59 | 164 | .988 | .028 | .021 |  |  |  |  |  |  |
| AI.2 | 711.96 | 181 | .986 | .029 | .024 | AI.2 vs. AI.1 | 82.37 | 17 | -.002 | .001 | .003 |
| AA.1 | 591.57 | 164 | .988 | .027 | .019 |  |  |  |  |  |  |
| AA.2 | 643.60 | 181 | .987 | .027 | .023 | AA.2 vs. AA.1 | 52.03 | 17 | -.001 | .000 | .004 |
| *Sample 2* |  |  |  |  |  |  |  |  |  |  |  |
| AS.1 | 525.50 | 170 | .993 | .023 | .018 |  |  |  |  |  |  |
| AS.2 | 547.65 | 187 | .993 | .022 | .018 | AS.2 vs. AS.1 | 22.15 | 17 | .000 | -.001 | .000 |
| AI.1 | 761.20 | 164 | .986 | .031 | .025 |  |  |  |  |  |  |
| AI.2 | 835.64 | 181 | .985 | .031 | .027 | AI.2 vs. AI.1 | 74.44 | 17 | -.001 | .000 | .002 |
| AA.1 | 626.90 | 164 | .989 | .027 | .019 |  |  |  |  |  |  |
| AA.2 | 665.44 | 181 | .988 | .026 | .021 | AA.2 vs. AA.1 | 38.54 | 17 | -.001 | -.001 | .002 |
| *Note.* AS = academic self-concept model; AI = academic anxiety model*;* AA = academic anxiety model; The numbers 1 and 2 in the model names indicate models with configural and metric invariance, respectively.  *df* = degrees of freedom; CFI = Comparative Fit Index; RMSEA= Root Mean Square Error of Approximation; SRMR = Standardized Root Mean Square Residual.  All χ^2^ goodness-of-fit tests were statistically significant at *p* < .001.  Values for Δχ^2^ were calculated according to the formula provided by Muthén (1998–2004, Formula 120) for the nested data structure. CFI values were calculated according to a null model (Model 0A) that is appropriate for investigating measurement invariance as suggested by Widaman and Thompson (2003). | | | | | | | | | | | |

| Table B2  *Academic Self-Concept: Standardized Factor Loadings and Latent Correlations as Obtained for the Metric Invariance Model (S1/S2)* | | | | | | | | | |
| --- | --- | --- | --- | --- | --- | --- | --- | --- | --- |
|  | Seventh grade | | | |  | Ninth grade | | | |
| Item | gASC | spMSC | spFSC | spGSC |  | gASC | spMSC | spFSC | spGSC |
| *Standardized factor loadings* | | | | | | | | | |
| SC_A1 | .65/.63 |  |  |  |  | .65/.66 |  |  |  |
| SC_A2 | .71/.71 |  |  |  |  | .73/.75 |  |  |  |
| SC_A3 | .80/.78 |  |  |  |  | .79/.80 |  |  |  |
| SC_M1 | .36/.40 | .79/.77 |  |  |  | .33/.37 | .81/.81 |  |  |
| SC_M2 | .44/.44 | .68/.70 |  |  |  | .42/.41 | .71/.73 |  |  |
| SC_M3 | .47/.47 | .79/.78 |  |  |  | .43/.43 | .80/.82 |  |  |
| SC_F1 | .20/.20 |  | .84/.85 |  |  | .19/.19 |  | .85/.87 |  |
| SC_F2 | .27/.28 |  | .79/.79 |  |  | .26/.27 |  | .81/.82 |  |
| SC_F3 | .30/.30 |  | .86/.87 |  |  | .29/.29 |  | .88/.88 |  |
| SC_G1 | .31/.28 |  |  | .78/.78 |  | .31/.28 |  |  | .78/.79 |
| SC_G2 | .43/.36 |  |  | .73/.73 |  | .42/.37 |  |  | .72/.73 |
| SC_G3 | .45/.40 |  |  | .78/.80 |  | .44/.40 |  |  | .77/.81 |
| *Correlations between factors* | | | | | | | | | |
| spMSC | 0 | - |  |  |  | 0 | - |  |  |
| spFSC | 0 | -.25/-.23 | - |  |  | 0 | -.18/-.21 | - |  |
| spGSC | 0 | -.26/-.28 | -.55/-.50 | - |  | 0 | -.29/-.27 | -.50/-.48 | - |
| *Note.* See Table A1 for descriptions of the individual items. gASC = general academic self-concept; spMSC = specific mathematics self-concept; spFSC = specific French self-concept; spGSC = specific German self-concept. All factor loadings were statistically significantly different from zero at *p* < .05. | | | | | | | | | |

| Table B3  *Academic Interest: Standardized Factor Loadings and Latent Correlations as Obtained for the Metric Invariance Model (S1/S2)* | | | | | | | | | |
| --- | --- | --- | --- | --- | --- | --- | --- | --- | --- |
|  | Seventh grade | | | |  | Ninth grade | | | |
| Item | gAINT | spMINT | spFINT | spGINT |  | gAINT | spMINT | spFINT | spGINT |
| *Standardized factor loadings* | | | | | | | | | |
| INT_A1 | .82/.82 |  |  |  |  | .82/.85 |  |  |  |
| INT_A2 | .62/.60 |  |  |  |  | .61/.62 |  |  |  |
| INT_A3 | .66/.65 |  |  |  |  | .70/.69 |  |  |  |
| INT_M1 | .50/.50 | .80/.82 |  |  |  | .48/.48 | .81/.83 |  |  |
| INT_M2 | .48/.45 | .57/.57 |  |  |  | .47/.45 | .60/.61 |  |  |
| INT_M3 | .39/.36 | .69/.71 |  |  |  | .38/.35 | .71/.72 |  |  |
| INT_F1 | .45/.47 |  | .79/.79 |  |  | .46/.48 |  | .78/.77 |  |
| INT_F2 | .42/.42 |  | .51/.52 |  |  | .44/.45 |  | .52/.53 |  |
| INT_F3 | .39/.38 |  | .78/.78 |  |  | .40/.40 |  | .77/.77 |  |
| INT_G1 | .45/.42 |  |  | .76/.78 |  | .45/.43 |  |  | .75/.75 |
| INT_G2 | .49/.43 |  |  | .57/.58 |  | .50/.47 |  |  | .57/.58 |
| INT_G3 | .42/.37 |  |  | .75/.78 |  | .42/.39 |  |  | .74/.77 |
| *Correlations between factors* | | | | | | | | | |
| spMINT | 0 | - |  |  |  | 0 | - |  |  |
| spFINT | 0 | -.14/-.11 | - |  |  | 0 | -.09/-.12 | - |  |
| spGINT | 0 | -.11/-.12 | -.32/-.29 | - |  | 0 | -.15/-.10 | -.28/-.27 | - |
| *Note.* See Table A1 for descriptions of the individual items. gAINT = general academic interest; spMINT = specific mathematics interest; spFINT = specific French interest; spGINT = specific German interest. All factor loadings were statistically significantly different from zero at *p* < .05. | | | | | | | | | |

| Table B4  *Academic Anxiety: Standardized Factor Loadings and Latent Correlations as Obtained for the Metric Invariance Model (S1/S2)* | | | | | | | | | |
| --- | --- | --- | --- | --- | --- | --- | --- | --- | --- |
|  | Seventh grade | | | |  | Ninth grade | | | |
| Item | gAANX | spMANX | spFANX | spGANX |  | gAANX | spMANX | spFANX | spGANX |
| *Standardized factor loadings* | | | | | | | | | |
| ANX_A1 | .57/.57 |  |  |  |  | .53/.54 |  |  |  |
| ANX_A2 | .85/.85 |  |  |  |  | .83/.86 |  |  |  |
| ANX_A3 | .79/.80 |  |  |  |  | .78/.81 |  |  |  |
| ANX_M1 | .45/.44 | .42/.44 |  |  |  | .40/.39 | .44/.44 |  |  |
| ANX_M2 | .66/.65 | .49/.54 |  |  |  | .63/.64 | .57/.59 |  |  |
| ANX_M3 | .72/.69 | .45/.50 |  |  |  | .68/.67 | .51/.54 |  |  |
| ANX_F1 | .26/.27 |  | .45/.49 |  |  | .23/.25 |  | .45/.46 |  |
| ANX_F2 | .67/.65 |  | .58/.60 |  |  | .65/.64 |  | .62/.61 |  |
| ANX_F3 | .61/.59 |  | .59/.62 |  |  | .58/.58 |  | .63/.63 |  |
| ANX_G1 | .41/.39 |  |  | .52/.49 |  | .38/.38 |  |  | .48/.47 |
| ANX_G2 | .60/.62 |  |  | .54/.54 |  | .60/.63 |  |  | .55/.54 |
| ANX_G3 | .66/.64 |  |  | .55/.56 |  | .65/.65 |  |  | .54/.56 |
| *Correlations between factors* | | | | | | | | | |
| spMANX | 0 | - |  |  |  | 0 | - |  |  |
| spFANX | 0 | -.24/-.18 | - |  |  | 0 | -.13/-.19 | - |  |
| spGANX | 0 | -.32/-.36 | -.40/-.43 | - |  | 0 | -.28/-.32 | -.35/-.31 | - |
| *Note.* See Table A1 for descriptions of the individual items. gAANX = general academic anxiety; spMANX = specific mathematics anxiety; spFANX = specific French anxiety; spGANX = specific German anxiety. All factor loadings were statistically significantly different from zero at *p* < .05. | | | | | | | | | |

# References

Browne, M. W., Cudeck, R. (1993). *Alternative ways of assessing model fit. In: Testing Structural Equation Models*, eds. K. Bollen & J. Long (Newbury Park, CA: Sage), 136–162.

Chen, F. F. (2007). Sensitivity of goodness of fit indexes to lack of measurement invariance. *Structural Equation Modeling*, 14, 464–504.

Cheung, G. W., & Rensvold, R. B. (2002). Evaluating goodness-of-fit indexes for testing measurement invariance. *Structural Equation Modeling*, 9, 233−255.

Hu, L. -T., & Bentler, P. M. (1998). Fit indices in covariance structure modeling: Sensitivity to underparameterized model misspecification. *Psychological Methods*, 3, 424−453.

Iacobucci, D. (2010). Structural equations modeling: Fit indices, sample size, and advanced topics. *Journal of Consumer Psychology*, 20, 90–98.

Khojasteh, J. & Lo, W.-J. (2015): Investigating the sensitivity of goodness-of-fit indices to detect measurement invariance in a bifactor model. Structural Equation Modeling: Multidisciplinary Journal, DOI: 10.1080/10705511.2014.937791

Little, T. (2013). *Longitudinal Structural Equation Modeling.* New York: Guilford Press.

Meade, A.W., Johnson, E. C., & Braddy, P.W. (2008). Power and sensitivity of alternative fit indices in tests of measurement invariance. *Journal of Applied Psychology*, 93, 568–592.

Muthén, B. O. (1998–2004). *Mplus technical appendices*. Los Angeles, CA: Muthén & Muthén.

Widaman, K. F., & Thompson, J. S. (2003). On specifying the null model for incremental fit indices in structural equation modeling. *Psychological Methods*, 8, 16−37.
