# Supplementary material for: Developmental Dynamics of General and School-Subject-Specific Components of Academic Self-Concept, Academic Interest, and Academic Anxiety
Source: Front Psychol. 2016 Mar 17;7:356. doi: 10.3389/fpsyg.2016.00356 (PMC4794478; doi:10.3389/fpsyg.2016.00356)
Supplement: Supplementary file 2 [file AppendixA.pdf]

Supplementary Material

Developmental Dynamics of General and School-Subject-Specific Components of Academic Self-Concept, Academic Interest, and Academic Anxiety

Katarzyna Gogol*, Martin Brunner, Franzis Preckel, Thomas Goetz, Romain Martin

*** Correspondence:** Katarzyna Gogol: katarzyna.gogol@fu-berlin.de

Table A1
Items Assessing Academic Self-Concepts, Academic Interests, and Academic Anxieties

General academic self-concept	
SC_A1	I do well on tests in most school subjects.
SC_A2	I learn things quickly in most school subjects.
SC_A3	I am good at most school subjects.
Subject-specific academic self-concepts	
SC_[S]1	[SUBJECT] is one of my best subjects.
SC_[S]2	I learn things quickly in [SUBJECT].
SC_[S]3	I am good at [SUBJECT].
General academic interest	
INT_A1	I am interested in most school subjects.
INT_A2	Most school subjects are important to me personally.
INT_A3	I enjoy most school subjects.
Subject-specific academic interests	
INT_[S]1	I am interested in [SUBJECT].
INT_[S]2	[SUBJECT] is important to me personally.
INT_[S]3	I enjoy [SUBJECT].
General academic anxiety	
ANX_A1	I am afraid of most school subjects.
ANX_A2	I get very nervous before tests in most school subjects.
ANX_A3	I am worried before tests in most school subjects.
Subject-specific academic anxieties	
ANX_[S]1	I am afraid of [SUBJECT] class.
ANX_[S]2	I get very nervous before tests in [SUBJECT].
ANX_[S]3	I am worried before tests in [SUBJECT].

Note. To assess subject-specific constructs, [SUBJECT] was replaced by mathematics, French, or German, respectively.
[a] Students responded to these items on a rating scale with four categories: *disagree*, *disagree somewhat*, *agree somewhat*, and *agree*.

Table A2

Sample 1: Descriptive Statistics, Correlations (below the Diagonal), and Covariances (above the Diagonal) for the Scale Scores Assessing Academic Self-Concept, Interest, and Anxiety

		Grade 7												Grade 9											
		SA	SM	SF	SG	IA	IM	IF	IG	AA	AM	AF	AG	SA	SM	SF	SG	IA	IM	IF	IG	AA	AM	AF	AG
Seventh grade	SA	-	.21	.14	.20	.17	.15	.09	.15	-.14	-.13	-.10	-.11	.12	.09	.04	.10	.06	.06	.01	.06	-.06	-.04	-.02	-.06
	SM	.42	-	-.03	.03	.14	.52	-.02	.03	-.16	-.42	.00	-.02	.09	.38	-.09	.00	.04	.29	-.07	-.01	-.08	-.23	.04	-.01
	SF	.26	-.04	-	-.21	.10	-.01	.54	-.13	-.03	.07	-.36	.20	.06	-.04	.53	-.23	.06	-.02	.38	-.13	.02	.08	-.23	.18
	SG	.39	.03	-.28	-	.12	.01	-.16	.51	-.14	-.03	.07	-.40	.10	-.06	-.27	.43	.03	-.05	-.21	.29	-.10	.00	.10	-.28
	IA	.43	.24	.17	.20	-	.27	.25	.26	-.03	-.03	-.02	.00	.09	.08	.06	.06	.18	.14	.12	.13	.02	.01	.02	.02
	IM	.29	.72	-.02	.01	.47	-	.14	.16	-.07	-.28	.04	.04	.07	.32	-.05	-.01	.12	.33	.03	.07	-.02	-.16	.05	.03
	IF	.18	-.02	.71	-.22	.44	.19	-	.05	.03	.10	-.23	.20	.05	-.01	.42	-.18	.14	.08	.40	-.03	.07	.08	-.15	.18
	IG	.29	.04	-.18	.70	.46	.22	.07	-	-.05	.03	.10	-.24	.08	-.03	-.16	.31	.13	.04	-.07	.32	-.03	.03	.09	-.15
	AA	-.28	-.23	-.04	-.20	-.05	-.10	.04	-.07	-	.47	.41	.41	-.09	-.10	.03	-.09	.00	-.03	.06	-.02	.26	.23	.15	.20
	AM	-.24	-.57	.09	-.04	-.05	-.38	.13	.04	.66	-	.29	.29	-.08	-.28	.10	-.03	.01	-.17	.11	.02	.22	.32	.09	.13
	AF	-.19	.00	-.47	.10	-.03	.05	-.31	.14	.59	.39	-	.19	-.07	-.02	-.25	.08	-.01	.01	-.15	.08	.17	.12	.29	.04
	AG	-.22	-.03	.26	-.55	-.01	.06	.28	-.33	.60	.39	.26	-	-.07	.01	.23	-.28	.02	.06	.21	-.13	.21	.13	.03	.34
Ninth grade	SA	.34	.18	.12	.19	.22	.15	.09	.16	-.18	-.15	-.13	-.13	-	.22	.14	.19	.20	.18	.11	.14	-.06	-.04	-.01	-.05
	SM	.16	.49	-.05	-.07	.13	.41	-.01	-.03	-.13	-.34	-.03	.02	.39	-	-.02	-.02	.16	.62	.01	-.01	-.07	-.39	.06	.06
	SF	.06	-.11	.64	-.33	.09	-.06	.52	-.20	.04	.12	-.31	.28	.24	-.02	-	-.23	.14	.03	.60	-.09	.07	.16	-.31	.26
	SG	.19	.00	-.30	.58	.10	-.01	-.24	.42	-.13	-.04	.10	-.38	.36	-.02	-.28	-	.13	.02	-.14	.50	-.07	.06	.17	-.32
	IA	.16	.07	.10	.06	.39	.21	.24	.22	.00	.01	-.02	.04	.48	.25	.22	.21	-	.28	.27	.27	.06	.06	.07	.07
	IM	.11	.37	-.03	-.06	.24	.43	.10	.05	-.05	-.22	.01	.07	.32	.73	.03	.02	.45	-	.16	.15	.03	-.22	.10	.11
	IF	.03	-.09	.48	-.27	.20	.03	.53	-.09	.09	.14	-.20	.27	.21	.01	.72	-.19	.45	.19	-	.07	.14	.19	-.14	.27
	IG	.12	-.01	-.17	.39	.23	.09	-.04	.44	-.03	.03	.10	-.18	.28	-.01	-.11	.66	.46	.19	.10	-	.04	.14	.18	-.12
	AA	-.13	-.12	.03	-.14	.04	-.02	.10	-.04	.40	.33	.26	.31	-.12	-.10	.10	-.10	.12	.04	.20	.06	-	.46	.36	.40
	AM	-.07	-.30	.10	.00	.02	-.20	.10	.04	.31	.41	.16	.17	-.08	-.47	.18	.08	.10	-.26	.23	.18	.65	-	.29	.30
	AF	-.04	.06	-.31	.13	.03	.07	-.20	.13	.22	.13	.39	.04	-.03	.08	-.38	.23	.12	.13	-.18	.24	.54	.37	-	.18
	AG	-.12	-.01	.24	-.39	.04	.05	.25	-.21	.29	.19	.05	.48	-.10	.07	.33	-.45	.13	.14	.37	-.17	.61	.40	.26	-
	M	3.03	2.95	2.74	2.98	3.09	3.03	2.87	2.91	2.08	1.98	1.98	1.88	2.86	2.60	2.51	2.84	2.70	2.65	2.59	2.61	2.13	2.22	2.09	1.88
	SD	.60	.85	.88	.86	.67	.85	.86	.85	.81	.87	.85	.85	.60	.93	.94	.87	.69	.91	.88	.86	.78	.90	.86	.83
	ω	.76	.90	.90	.89	.74	.86	.86	.87	.80	.82	.83	.83	.77	.90	.91	.89	.76	.87	.87	.87	.79	.83	.82	.82

Note. Scale scores for academic self-concepts, interests, and anxieties were computed as the mean of the respective item scores. Reliability estimates were calculated as coefficient ω (McDonald, 1999) and were based on congeneric first-order factor models. ω can be interpreted in the same way as any other reliability coefficient, with values that can range from 0 (no reliability) to 1 (perfect reliability). SA = academic self-concept; SM = mathematics self-concept; SF = French self-concept; SG = German self-concept; IA = academic interest; IM = mathematics interest; IF = French interest; IG = German interest; AA = academic anxiety; AM = mathematics anxiety; AF = French anxiety; AG = German anxiety.

Table A3

Sample 2: Descriptive Statistics, Correlations (below the Diagonal), and Covariances (above the Diagonal) for the Scale Scores Assessing Academic Self-Concept, Interest, and Anxiety

		Grade 7												Grade 9											
		SA	SM	SF	SG	IA	IM	IF	IG	AA	AM	AF	AG	SA	SM	SF	SG	IA	IM	IF	IG	AA	AM	AF	AG
Seventh grade	SA	-	.22	.13	.17	.16	.15	.09	.11	-.15	-.16	-.10	-.12	.13	.11	.04	.09	.06	.08	.01	.04	-.08	-.07	-.04	-.07
	SM	.43	-	-.03	.00	.12	.52	-.03	.00	-.17	-.47	.00	-.01	.11	.41	-.05	-.03	.04	.29	-.06	-.05	-.10	-.27	.00	.00
	SF	.25	-.03	-	-.20	.11	.01	.53	-.13	-.02	.06	-.37	.21	.07	-.05	.52	-.21	.07	-.02	.38	-.13	.02	.09	-.25	.16
	SG	.35	-.01	-.27	-	.10	-.02	-.14	.47	-.15	-.01	.09	-.43	.07	-.05	-.27	.40	.02	-.04	-.19	.26	-.09	.01	.10	-.28
	IA	.41	.21	.19	.18	-	.24	.24	.22	-.01	-.02	-.01	.01	.08	.06	.06	.05	.15	.11	.11	.11	.02	.02	.00	.02
	IM	.30	.71	.01	-.03	.44	-	.14	.11	-.08	-.30	.03	.05	.08	.32	.00	-.04	.11	.31	.05	.01	-.04	-.17	.02	.05
	IF	.18	-.04	.71	-.20	.43	.19	-	.04	.03	.10	-.24	.20	.04	-.04	.40	-.15	.14	.04	.38	-.03	.06	.11	-.15	.15
	IG	.23	.00	-.18	.68	.41	.16	.05	-	-.05	.05	.12	-.25	.05	-.05	-.15	.27	.09	.02	-.06	.28	-.01	.06	.10	-.13
	AA	-.31	-.24	-.03	-.21	-.02	-.12	.05	-.07	-	.49	.42	.44	-.10	-.13	.04	-.10	.01	-.05	.09	-.01	.30	.27	.18	.23
	AM	-.29	-.60	.07	-.02	-.04	-.39	.13	.06	.65	-	.30	.29	-.10	-.31	.08	-.02	.01	-.18	.12	.05	.26	.38	.14	.15
	AF	-.18	.00	-.48	.12	-.01	.05	-.32	.16	.57	.37	-	.18	-.08	-.02	-.27	.08	-.01	.02	-.16	.09	.20	.13	.32	.06
	AG	-.23	-.01	.27	-.59	.01	.07	.26	-.34	.61	.37	.24	-	-.06	.01	.24	-.31	.03	.05	.22	-.15	.24	.17	.05	.36
Ninth grade	SA	.37	.22	.14	.15	.20	.16	.09	.10	-.21	-.18	-.16	-.13	-	.21	.14	.16	.17	.16	.10	.11	-.09	-.07	-.04	-.06
	SM	.19	.50	-.06	-.07	.10	.40	-.05	-.06	-.16	-.37	-.02	.01	.39	-	-.03	-.03	.14	.62	-.02	-.02	-.09	-.43	.06	.06
	SF	.06	-.06	.64	-.34	.11	.00	.50	-.20	.05	.10	-.33	.30	.26	-.03	-	-.22	.15	.01	.60	-.10	.06	.14	-.32	.25
	SG	.18	-.04	-.29	.57	.08	-.06	-.22	.39	-.15	-.02	.11	-.42	.33	-.03	-.28	-	.10	.00	-.15	.44	-.09	.05	.13	-.35
	IA	.14	.06	.13	.03	.36	.19	.24	.17	.03	.02	-.02	.06	.44	.23	.24	.19	-	.26	.27	.24	.05	.05	.04	.06
	IM	.14	.37	-.03	-.06	.20	.40	.06	.03	-.06	-.22	.02	.07	.31	.73	.02	.00	.44	-	.13	.13	.00	-.24	.11	.11
	IF	.02	-.07	.49	-.26	.20	.07	.51	-.08	.12	.15	-.20	.29	.20	-.02	.73	-.20	.47	.17	-	.07	.13	.19	-.15	.25
	IG	.08	-.07	-.18	.38	.20	.01	-.04	.41	-.02	.07	.12	-.21	.23	-.03	-.13	.64	.45	.17	.10	-	.03	.12	.15	-.15
	AA	-.17	-.15	.03	-.14	.03	-.06	.08	-.01	.47	.37	.29	.35	-.19	-.13	.08	-.14	.09	.00	.19	.04	-	.46	.37	.41
	AM	-.13	-.34	.11	.01	.03	-.22	.14	.08	.36	.47	.17	.21	-.14	-.51	.17	.06	.08	-.29	.24	.16	.65	-	.26	.28
	AF	-.08	.00	-.33	.14	.00	.02	-.21	.14	.26	.18	.43	.06	-.09	.07	-.41	.18	.07	.14	-.21	.22	.56	.34	-	.19
	AG	-.14	.00	.22	-.40	.04	.07	.21	-.19	.34	.20	.08	.50	-.12	.07	.32	-.51	.11	.14	.35	-.22	.63	.38	.27	-
	M	3.01	2.94	2.76	2.95	3.14	3.07	2.97	2.96	2.12	2.01	1.99	1.92	2.89	2.64	2.54	2.88	2.78	2.71	2.62	2.69	2.16	2.22	2.09	1.89
	SD	.59	.86	.88	.84	.65	.84	.85	.82	.83	.90	.88	.87	.58	.93	.94	.83	.66	.90	.87	.82	.78	.90	.84	.83
	ω	.75	.90	.91	.88	.74	.86	.86	.85	.81	.83	.84	.83	.78	.92	.92	.89	.77	.88	.87	.87	.81	.84	.82	.83

Note. Scale scores for academic self-concepts, interests, and anxieties were computed as the mean of the respective item scores. Reliability estimates were calculated as coefficient ω (McDonald, 1999) and were based on congeneric first-order factor models. ω can be interpreted in the same way as any other reliability coefficient, with values that can range from 0 (no reliability) to 1 (perfect reliability). SA = academic self-concept; SM = mathematics self-concept; SF = French self-concept; SG = German self-concept; IA = academic interest; IM = mathematics interest; IF = French interest; IG = German interest; AA = academic anxiety; AM = mathematics anxiety; AF = French anxiety; AG = German anxiety.
